# AAV serotype 8-mediated liver specific GNMT expression delays progression of hepatocellular carcinoma and prevents carbon tetrachloride-induced liver damage

**DOI:** 10.1038/s41598-018-30800-3

**Published:** 2018-09-14

**Authors:** Cheng-Chieh Fang, Ching-Fen Wu, Yi-Jen Liao, Shiu-Feng Huang, Marcelo Chen, Yi-Ming Arthur Chen

**Affiliations:** 1 Center for Infectious Disease and Cancer Research (CICAR), Kaohsiung Medical University, Kaohsiung, Taiwan; 2 National Mosquito-Borne Diseases Control Research Center, National Health Research Institutes, Miaoli, Taiwan; 3 School of Medical Laboratory Science and Biotechnology, College of Medical Science and Technology, Taipei Medical University, Taipei, Taiwan; 4 Institute of Molecular and Genomic Medicine, National Health Research Institutes, Miaoli, Taiwan; 5 Department of Urology, Mackay Memorial Hospital, Taipei, Taiwan; 6 School of Medicine, Mackay Medical College, New Taipei City, Taiwan; 7 Institute of Biomedical Sciences, National Sun Yat-sen University, Kaohsiung, Taiwan; 8 Department of Microbiology and Immunology, Institute of Medical Research and Institute of Clinical Medicine, College of Medicine, Kaohsiung Medical University, Kaohsiung, Taiwan

## Abstract

Glycine N-methyltransferase (GNMT) is abundantly expressed in normal livers and plays a protective role against tumor formation. GNMT depletion leads to progression of hepatocellular carcinoma (HCC). In this study, we investigated the activity of ectopic GNMT delivered using recombinant adeno-associated virus (AAV) gene therapy in mouse models of liver cirrhosis and HCC. Injection of AAV serotype 8 (AAV8) vector carrying the GNMT gene (AAV8-GNMT) in *Gnmt*^−/−^ mice increased GNMT expression and downregulated pro-inflammatory responses, resulting in reduced liver damage and incidence of liver tumors. Moreover, AAV8-GNMT resulted in the amelioration of carbon tetrachloride (CCl_4_)-induced liver fibrosis in BALB/c mice. We showed that AAV8-GNMT protected hepatocytes from CCl_4_-induced liver damage.  AAV8-GNMT significantly attenuated the levels of pro-fibrotic markers and increased efficiency of hepatocyte proliferation. These results suggest that correction of hepatic GNMT by gene therapy of AAV8-mediated gene enhancement may provide a potential strategy for preventing and delaying development of liver diseases.

## Introduction

Liver damage and persistent inflammation because of environmental toxins, alcohol abuse, non-alcoholic fatty liver syndrome and chronic viral infection lead to liver cirrhosis and eventually to the development of hepatocellular carcinoma (HCC) within decades^1,2^. Due to the limitations of current therapies, liver cirrhosis and HCC remain highly prevalent and cause important public health issues worldwide^3,4^. Gene therapy is a powerful therapeutic tool. Genetic modification is achieved by introducing genetic materials into target cells to regulate biological functions^5^. The liver represents a preferred target for gene therapy due to its unique anatomy. Its duel blood supply system and specific fenestrated endothelium lining along the sinusoids efficiently allow high level of gene vectors to target hepatocytes, making liver-directed gene therapy favorable for the treatment of liver monogenic diseases^6,7^. Challenges arising from liver-directed gene therapy involve efficient hepatocyte transduction, sustainability of gene expression and vector-induced immune responses. The adeno-associated virus (AAV) overcomes these obstacles and exhibits ideal characteristics as a promising viral vector for gene transfer^7^. In addition, AAV infects both dividing and quiescent cells *in vivo* and persists as an extrachromosomal state without integrating into host chromosome, reducing the possibility of insertional mutagenesis^8^. Among the investigated AAV serotype vectors, AAV8 exerts higher affinity to hepatocytes and presumably transduce more than 90–95% of hepatocytes via intraportal vein injection^9,10^. A clinical study led by Nathwani *et al*. has demonstrated that the AAV8-FIX vector could be successfully delivered to the liver and appeared to clinically benefit patients with hemophilia B in terms of safety^11^. Utilization of AAV8 vector targeting hepatic fibrosis and HCC has been investigated *in vivo*^12–14^.

Glycine N-methyltransferase (GNMT) is a multiple functional protein that regulates the cellular pool of methyl groups by controlling the ratio of S-adenosylmethionine (SAM) to S-adenosylhomocysteine (SAH)^15,16^. GNMT is highly expressed in the peri-portal region of the normal liver^17^ and has been depicted for its function in detoxification against environmental carcinogens, such as polycyclic aromatic hydrocarbons, benzo(a)pyrene (BaP) and aflatoxin B1, via translocation from the cytoplasm to the nucleus^18–20^. Furthermore, Chen *et al*. analyzed sets of liver tissues from HCC patients and found that GNMT expression was reduced or undetectable in tumor cells, while it remained abundant in non-tumor ones^21^. These lines of evidences suggest a protective role of GNMT and has led to investigations on mechanisms of downregulation of the GNMT gene. Ours and Luca *et al*.’s groups generated *Gnmt*^−/−^ mice and both of the knockout models developed liver injury and HCC spontaneously, indicating the essential role of GNMT in tumor suppression in the liver^22–25^. During tumor formation in GNMT deficient mice, GNMT acted as a negative regulator in the mitogen-activated protein kinase (MAPK), wingless-type MMTV integration site (Wnt) and Janus kinase and signal transducer and activator of transcription (JAK-STAT) signaling pathways^23,26^. A recent study revealed that GNMT depletion reduced PREX2 ubiquitination, leading to activation of PI3K/AKT pathway and tumorigenesis^27^. Based on these results, we hypothesized that overexpression of GNMT restored liver pathogenesis induced by GNMT deficiency. In this study, we aimed to develop an AAV8 vector carrying human GNMT gene and determined whether GNMT compensation reduced HCC incidence in *Gnmt*^−/−^ mice. The effects of GNMT enhancement were examined using the BALB/c mouse model of carbon tetrachloride (CCl_4_)-induced liver injury.

## Results

### The pAAV8-GNMT construct and production of AAV8 vector carrying GNMT

**Figure 1:**
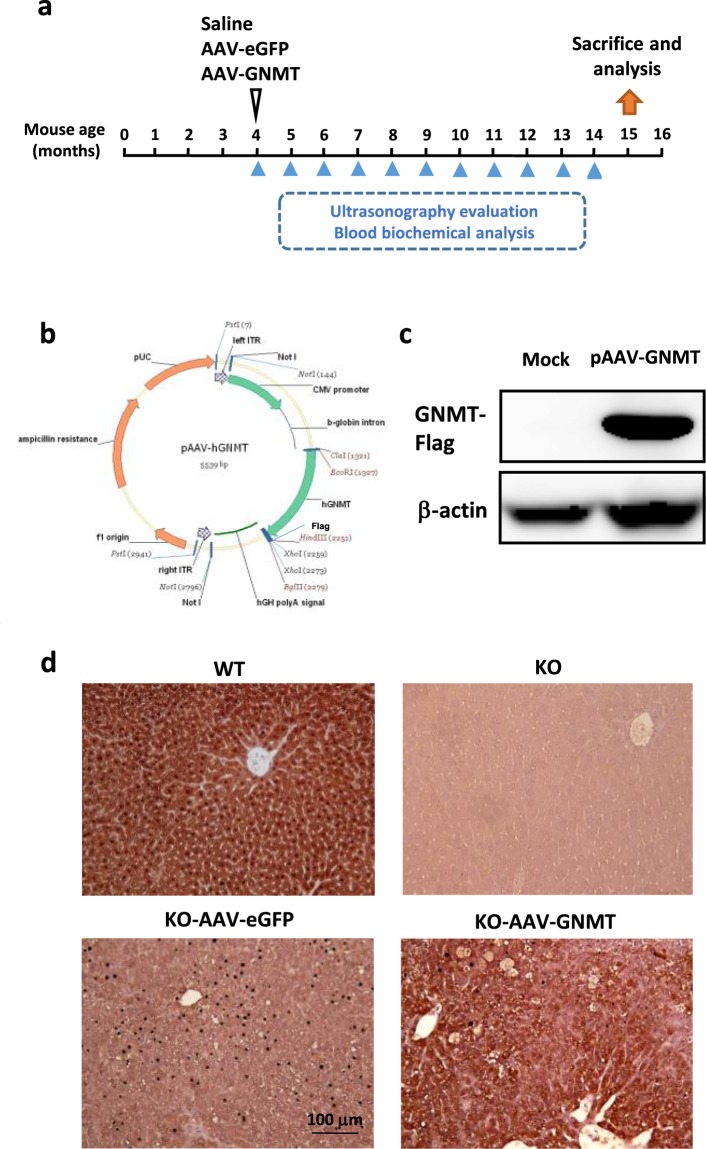
AAV8 plasmid bearing the GNMT gene and GNMT protein expression in the liver tissues. (**a**) Representative diagram of AAV8 injection in *Gnmt*^−/−^ (KO) mice. (**b**) The schematic construct of pAAV8-GNMT. (**c**) GNMT expression of 293T cells transfected with pAAV8-GNMT was measured by western blotting. The original picture of western blotting was provided in Fig. S1. (**d**) Staining for GNMT was performed on liver sections of WT, KO, KO-AAV8-eGFP and KO-AAV8-GNMT mice by immunohistochemical assessment.

Human GNMT gene was fused with Flag sequence in the C-terminus of GNMT gene by PCR and the Flag-tag GNMT gene was inserted into the backbone vector AAV8 (Fig. 1b). After transfecting pAAV8-GNMT in 293T cells, GNMT expression was confirmed by western blotting (Fig. 1c). This data indicated that the GNMT expressing cassette could be used in mammalian cells.

Immunohistochemical analysis revealed that GNMT was abundantly expressed in the livers of wild type (WT) mice, while the staining was undetectable in the livers of *Gnmt*^−/−^ (KO) mice and *Gnmt*^−/−^ mice with AAV8-eGFP injection (Fig. 1d). As shown in Fig. 1d, AAV8-mediated GNMT was successfully overexpressed in the liver of *Gnmt*^−/−^ mice, and GNMT overexpression potentially lasted up to 11 months. According to the distribution of GNMT, AAV8-mediated GNMT was expressed around the central vein, particularly in the area where the lesions existed.

After a 40-week follow-up, we did not find significant adverse effects in mice with hepatic AAV8-mediated GNMT. Serum biochemical values of alanine aminotransferase (ALT), triglyceride and cholesterol, which are used to detect liver damage and liver function in lipid metabolism, were within the normal range of reference values in both AAV8-treated groups. Histopathological analysis of liver sections showed normal and regular arrangement of parenchyma. These results showed that hepatocytes and liver function were not affected by AAV8-mediated GNMT overexpression, suggesting the long-term safety of AAV8-GNMT in the treated mice (Data not shown).

### Hepatic AAV8-mediated GNMT reduced liver injury and inflammation in *Gnmt*^−/−^ mice

**Figure 2:**
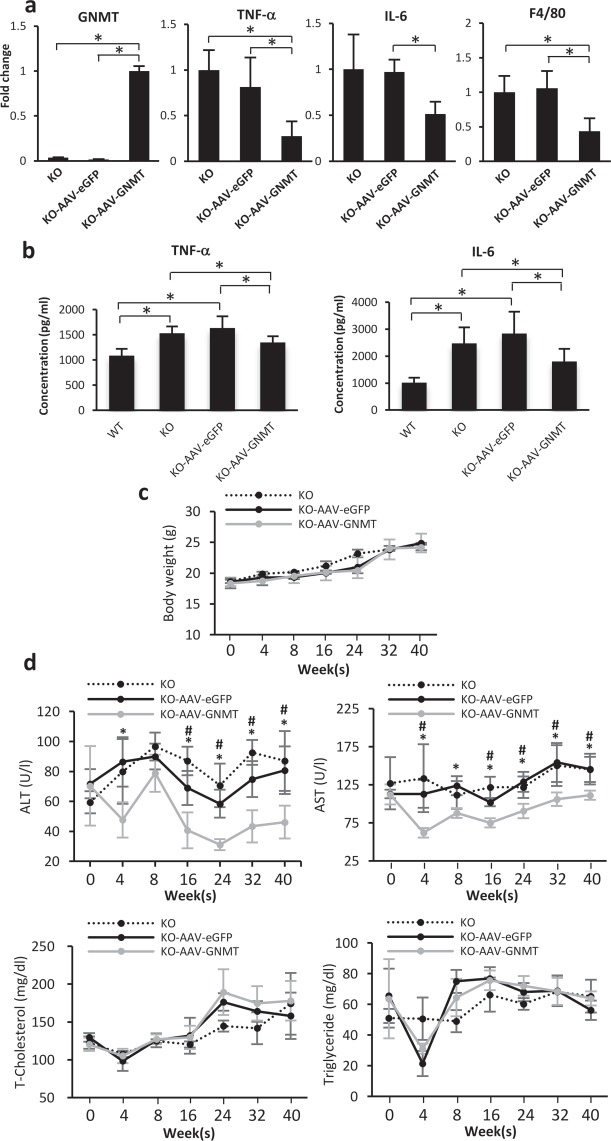
Evaluation of liver injury in AAV8-injected *Gnmt*^−/−^ mice. (**a**) Levels of GNMT and pro-inflammatory genes including TNF-α, IL-6 and F4/80 were measured by qPCR. *p < 0.05. (**b**) Serum levels of TNF-α and IL-6 were detected by ELISA analysis. *p < 0.05. (**c**) Body weight and (**d**) the values of serum biochemistry including ALT, AST, triglyceride and cholesterol were examined at indicated time points. *p < 0.05 when KO-AAV8-GNMT group is compared with KO-AAV8-eGFP. ^#^p < 0.05 when KO-AAV8-GNMT group is compared with KO.

Our previous studies demonstrated that *Gnmt*^−/−^ mice develop chronic hepatitis with significant elevation of serum level of ALT^25^. Therefore, we investigated the feasibility of the AAV8 vector carrying the GNMT gene in mice with GNMT deficiency-induced liver injury. Significant GNMT overexpression in AAV8-GNMT treated *Gnmt*^−/−^ mice was detected after 11 months of experiment, confirming the long-tern expression of AAV8-mediated GNMT expression in GNMT deficient mice (Fig. 2a). Chronic inflammation was reported to increase the risk and accelerate the development of liver cancer via induction of inflammatory mediators including interleukin-6 (IL-6) and tumor necrosis factor-α (TNF-α)^28^. The mRNA expression levels of TNF-α, IL-6 and a specific mouse macrophage marker F4/80 were lower in livers of AAV8-GNMT treated *Gnmt*^−/−^ mice as compared to AAV8-eGFP treated ones (Fig. 2a). In addition, the presence of GNMT significantly reduced the elevated serum levels of TNF-α and IL-6 in *Gnmt*^−/−^ and AAV8-eGFP treated *Gnmt*^−/−^ mice (Fig. 2b).The serum levels of ALT and aspartate aminotransferase (AST) of *Gnmt*^−/−^ mice treated with AAV8-GNMT were lower than that in *Gnmt*^−/−^ mice treated with AAV8-eGFP (Fig. 2d). Meanwhile, there was no significant difference in body weight (Fig. 2c), serum levels of triglyceride and cholesterol between AAV8-eGFP and AAV8-GNMT groups (Fig. 2d). These results showed that hepatic AAV8-GNMT reduced liver injury and inflammation in *Gnmt*^−/−^ mice.

### Hepatic AAV8-mediated GNMT delayed tumor formation in *Gnmt*^−/−^ mice

**Figure 3:**
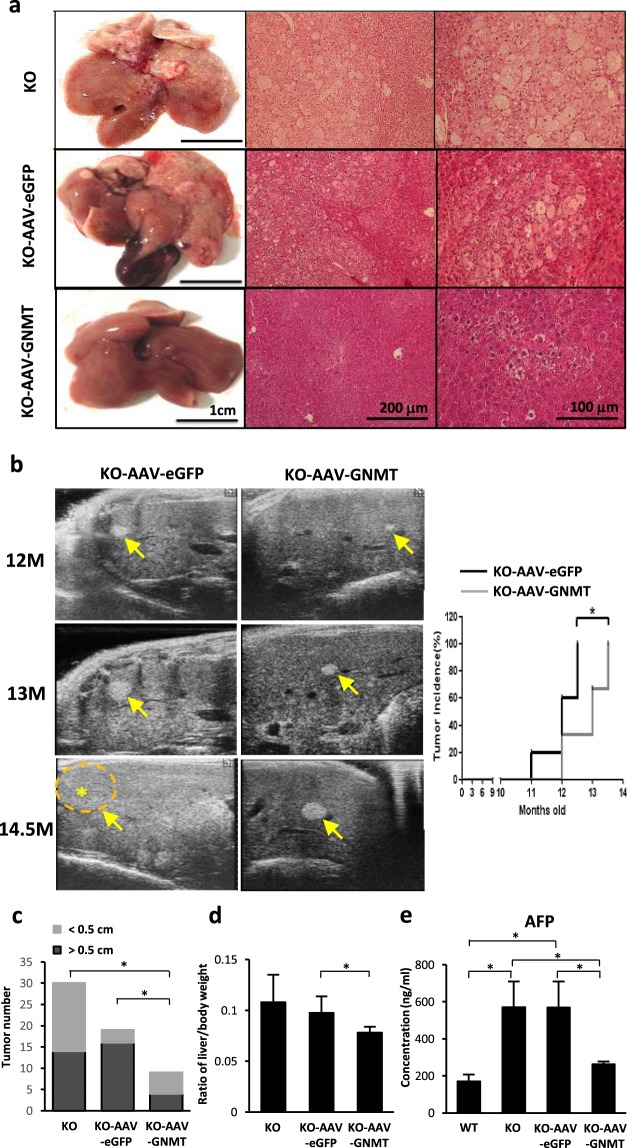
Tumor formation in the liver of AAV8-injected *Gnmt*^−/−^ mice. (**a**) Histological analysis of liver tissue sections was performed by hematoxylin and eosin (H&E) staining. (**b**) The development of liver tumors was monitored by B-mode ultrasonography. Star and arrow symbols indicate the location of tumor. Quantification of tumor incidence was analyzed. (**c**) Tumor formation and (**d**) the ratio of liver weight divided to body weight were evaluated. (**e**) Serum level of AFP was detected by ELISA analysis. *p < 0.05 when considered to be statistically significant.

Our previous data showed that 100% of female *Gnmt*^−/−^ mice developed HCC spontaneously^26^. In this study, tumor nodules from *Gnmt*^−/−^ and AAV8-eGFP treated *Gnmt*^−/−^ mice had irregular infiltration borders, perinuclear vacuoles, pleomorphic tumor cells, fatty change and regional inflammation, while AAV8-GNMT administration inhibited the progression of liver cancer in *Gnmt*^−/−^ mice (Fig. 3a). Ultrasonography showed that tumors were less developed in AAV8-GNMT injected *Gnmt*^−/−^ mice. Liver nodules were detected in all 5 AAV8-eGFP treated *Gnmt*^−/−^ mice, but only in 2 of 6 (33.3%) AAV8-GNMT treated *Gnmt*^−/−^ mice at the age of 12.5 months. However, all AAV8-GNMT injected *Gnmt*^−/−^ mice developed tumors at 13.5 months (Fig. 3b). AAV8-GNMT treatment decreased tumor size. The existing tumors larger than 0.5 cm were significantly decreased in AAV8-GNMT injected *Gnmt*^−/−^ mice (Fig. 3c). We also observed lower ratio of liver weight/body weight in AAV8-GNMT treated mice compared to AAV8-eGFP treated ones. (Figure 3d). α-fetoprotein (AFP) is a tumor associated protein and the well-known serum biomarker used for HCC surveillance^29^. *Gnmt*^−/−^ mice and AAV8-eGFP treated *Gnmt*^−/−^ mice showed significant AFP induction in mice serum, while AAV8-GNMT reduced its level close to basal line (Fig. 3e). These results indicated that AAV8-GNMT treatment delayed the progression of tumor growth, but its effects were not sufficient to halt the formation of tumors in *Gnmt*^−/−^ mice.

### Hepatic AAV8-mediated GNMT reduced CCl4-induced fibrosis

**Figure 4:**
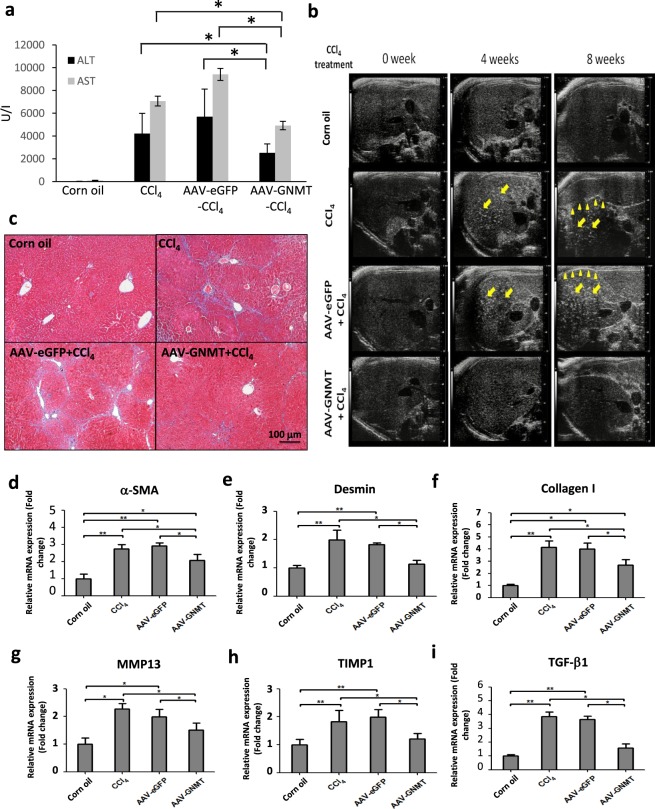
CCl_4_-induced liver fibrosis after AAV8 injection. (**a**) Serum levels of ALT and AST were measured on the second day of CCl_4_ injection in the sixth week. (**b**) Liver fibrosis of experimental mice was examined by ultrasonography. The arrow indicates heterogeneous parenchymal echogenicity and the arrowhead indicates irregular surface of liver. (**c**) Representative figures of the Masson’s trichrome-stained mouse livers. The mRNA levels of (**d**) α-SMA, (**e**) desmin, (**f**) collagen I, (**g**) MMP13, (**h**) TIMP1, (**i**) TGF-β1 were measured by qPCR. **p < 0.01, *p < 0.05 when considered to be statistically significant.

In the injured area with oxygen, CCl_4_ is converted to CCl_3_-OO* radicals, which ultimately results in damage of membrane integrity and cellular necrosis. Administration of CCl_4_ causes cycles of tissue damage, inflammation and repair, leading to fibrosis^30^. Next, we investigated whether hepatic AAV8-mediated GNMT expression exerted protective effects against CCl_4_-induced hepatic damage and fibrosis in wild-type BALB/c mice. We checked ALT and AST levels on the second day of injections and the result indicated that CCl_4_ induced severe damage in all groups in the second and fourth weeks (data not shown). Notably, the mice with pre-treatment of AAV8-GNMT showed significantly lower levels of ALT and AST in the sixth week in comparison with the CCl_4_ and AAV8-eGFP/CCl_4_ groups (Fig. 4a). Livers from CCl_4_ treatment alone or with AAV8-eGFP injection showed increased heterogeneous parenchymal echogenicity and irregular liver surface, and AAV8-GNMT injection prior to CCl_4_ treatment revealed less echogenicity and regular hepatic surface (Fig. 4b). Consistent with the ultrasonographic findings, livers from AAV8-eGFP/CCl_4_ group exhibited significant collagen deposition by Masson’s trichrome staining. Collagen accumulation was significantly decreased in the AAV8–GNMT/CCl_4_ group (Fig. 4c).

Liver fibrosis is characterized by hepatic stellate cell (HSC) activation and accumulation of extracellular matrix (ECM) and collagen^31^. Thus, the expression of α-smooth muscle actin (α-SMA), a typical marker of activated HSCs^32^, was assessed to evaluate the effect of hepatic AAV8-mediated GNMT on HSC activation during hepatic fibrosis. Desmin is another specific marker of activated HSCs. Analysis of qPCR showed that AAV8–GNMT/CCl_4_ mice had significantly lower expression of α-SMA and desmin than either the AAV8-eGFP/CCl_4_ or CCl_4_ mice, whereas significantly higher expression than the corn oil group (Fig. 4d,e). These results suggested that AAV8–GNMT may inhibit CCl_4_-induced HSC activation in mice.

**Figure 5:**
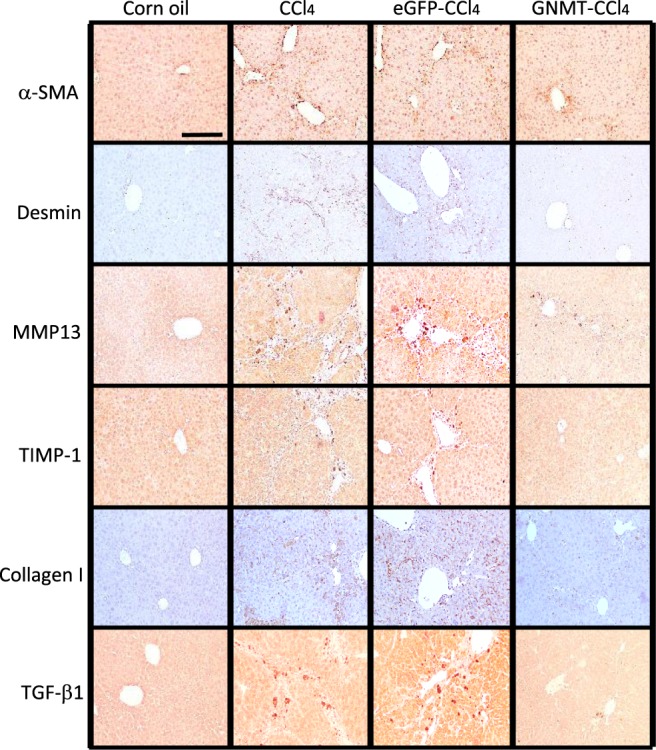
Expression of liver fibrotic markers in the AAV8-injected mice. The levels of α**-**SMA, desmin, collagen I, MMP13, TIMP1 and TGF-β1 were detected on the liver sections using primary antibodies by immunohistochemistry staining. Scale bar: 100 μm.

**Figure 6:**
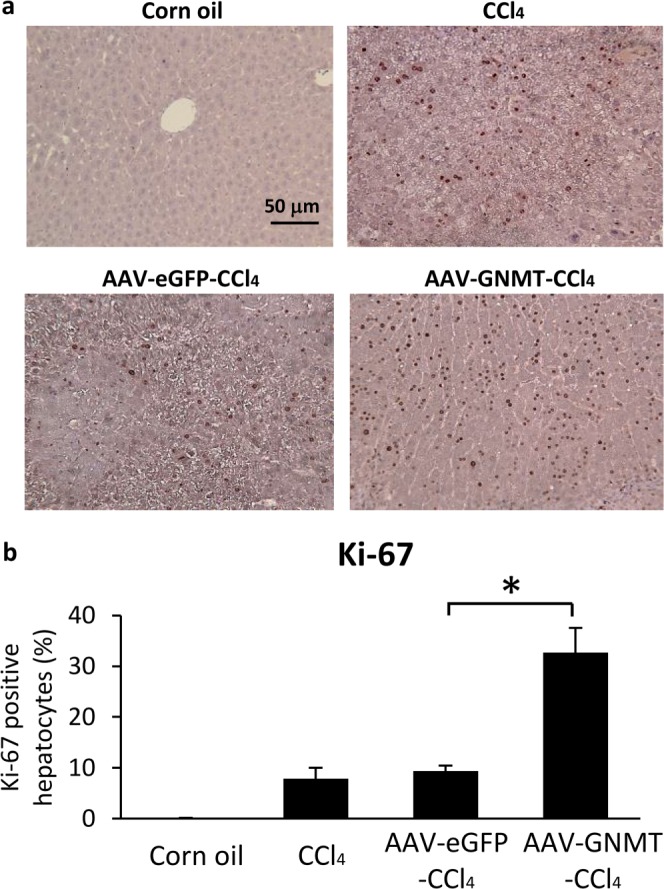
AAV8-GNMT vector promoted liver regeneration in CCl_4_-induced damage. (**a**) Ki-67-positive hepatocytes were detected by immunohistochemistry. (**b**) Ki-67-positive hepatocytes and total hepatocytes were calculated in each mouse liver section by counting 10 random fields at 200 magnification. The percentage of replicating Ki67-positive hepatocytes was determined. *p < 0.05 when considered to be statistically significant.

During chronic liver inflammation, damaged hepatocytes, activated Kupffer cells and endothelial cells release transforming growth factor (TGF)-β and other factors to activate quiescent HSCs. The activated HSCs produce large amounts of ECM components, such as collagen type I resulting in fibrotic change in the liver. The expression of collagen I has been shown to increase during the development of liver cirrhosis^33^. Tissue inhibitor of metalloproteinases-1 (TIMP1), which prevents the degradation of the ECM by inhibiting members of a large family of MMPs, has also been shown to increase in the development of liver fibrosis both in murine experimental models and human samples^34,35^. As CCl_4_-stimulated HSC activation and collagen deposition were ameliorated by AAV8-GNMT administration, we further investigated the mRNA expression of the specific mediators involved in the progression of liver fibrosis. Quantitative PCR showed that CCl_4_-induced expression of TGF-β1, TIMP1, collagen I and MMP13 was significantly inhibited in the AAV8-GNMT injected group (Fig. 4f–i). Next, expression of the aforementioned important markers was further verified by immunohistochemistry staining. As shown in Fig. 5, strong positive reactions to the anti-α-SMA, desmin, TGF-β1, TIMP1, collagen I and MMP13 antibodies were detected in the CCl_4_ and CCl_4_-AAV8-eGFP livers. Expression of these proteins in CCl_4_-AAV8-GNMT livers was decreased (Fig. 5). Damage of hepatocytes triggers not only wound healing, which leads to liver fibrosis if fibrotic matrix is continuously accumulated, but also liver regeneration. Moreover, hepatocyte proliferation prevents normal parenchyma from being replaced by fibrotic matrix^36^. We hypothesized that GNMT overexpression played a role in hepatocyte proliferation in CCl_4_-induced liver injury. The cell proliferation marker Ki-67^37^, which is expressed only in the nuclei of actively proliferating cells, was detected one day after the first CCl_4_ injection. As shown in Fig. 6, Ki-67 was significantly upregulated in AAV8-GNMT-CCl_4_ treated mouse livers in comparison with AAV8-eGFP-CCl_4_ and CCl_4_ groups (Fig. 6). Taken together, these results collectively demonstrated that AAV8–GNMT inhibited CCl_4_-induced hepatic fibrosis.

## Discussion

GNMT, a tumor suppressor gene, has been abundantly explored for its role in the progression of HCC. *Gnmt*^−/−^ mice gradually progresses to steatohepatitis, cirrhosis and HCC; therefore, these mice serve as a useful tool to uncover underlying molecular mechanisms and to develop therapeutic agents against the pathogenesis of HCC. Taking advantage of AAV’s efficient transduction and inability to induce significant immune responses and side effects, we used hepatotropic AAV serotype 8 to deliver human GNMT into mouse hepatocytes to achieve sustained GNMT expression in *Gnmt*^−/−^ mice. According to our previous report, GNMT depletion dysregulates the expression of genes related to detoxification and anti-oxidation, leading to oxidative stress and liver damage^38^. In addition, GNMT modulates immune responses in different mouse models. GNMT depletion leads to activation of natural killer (NK) cells and upregulation of T helper (Th)1- over Th2-related cytokines and TNF-related apoptosis-inducing ligand (TRAIL), contributing to pro-inflammatory response in the livers^39^. Chen *et al*. demonstrated that GNMT regulates oxidized low-density lipoprotein-mediated cholesterol accumulation and TNF-induced inflammation response in foamy macrophages, and additionally, genetic depletion of GNMT provokes pathogenesis of atherosclerosis in apolipoprotein E deficient mice^40^. In the mouse model of dextran sulfate sodium (DSS)-induced colitis, *Gnmt*^−/−^ mice are more susceptible to DSS-induced colonic inflammation^41^. These reports suggest that GNMT exerts anti-inflammatory effects against induction of innate immune responses. Here, we demonstrated that a single administration of AAV8-GNMT vector exerted long-term GNMT overexpression in the livers of *Gnmt*^−/−^ mice, which protects GNMT deficient hepatocytes from liver injuries validated by significant downregulation in ALT and AST levels. Macrophages and monocytes are important sources of pro-inflammatory cytokines in the liver and key players in the progression of hepatic inflammation. The presence of AAV8-GNMT decreased the levels of F4/80, TNF-α and IL-6, suggesting inhibition of inflammatory responses and ultimately leading to delayed development of HCC.

Hepatic AAV8-mediated GNMT expression exerted only partial inhibitory effects and was not able to completely abolish liver pathogenesis in *Gnmt*^−/−^ mice. First, this phenomenon might be attributed to the time point during which the mice were administrated with AAV8-GNMT. Principally, mice express GNMT at the age of 3 weeks, namely at the stage of weaning^42^. In this study, livers of *Gnmt*^−/−^ mice at the age of 4 months developed chronic steatohepatitis and tumor-associated signaling pathways stimulated by GNMT deficiency were initiated. Therefore, GNMT gene supplementation may delay, but might not be sufficient to correct the pathogenesis entirely. Second, the AAV8-GNMT vector was not evenly distributed in the *Gnmt*^−/−^ livers. The AAV8 vector is capable of efficiently transducing both normal and damaged liver tissues^43,44^. In addition, AAV8 transduces hepatocytes surrounding central veins more efficiently than the ones around portal areas through portal vein, tail vein or intraperitoneal injection^45^. In agreement with the reported results, AAV8-GNMT vectors were primarily expressed in the lesions of the pericentral regions. As previously mentioned, GNMT is expressed abundantly in the periportal regions. Therefore, damages induced by GNMT deficiency potentially accumulated in the area where AAV8-GNMT vectors were less expressed, implying a limitation of AAV8-GNMT administration in the *Gnmt*^−/−^ model. Third, loss of AAV8-mediated GNMT expression may occur during the tumor formation in the livers of *Gnmt*^−/−^ mice. Thus, additional AAV8-GNMT injection to boost GNMT expression may be necessary in the *Gnmt*^−/−^ model. Finally, human GNMT may be less efficient in the mouse model due to differences of amino acid sequences between species.

Next, we hypothesized that pre-treatment of AAV8-GNMT prevented liver damage by CCl_4_. The increased ALT and AST levels upon CCl_4_ treatment, which were seen in the AAV8-GNMT and AAV8-eGFP mice, indicated that the AAV8-mediated GNMT gene enhancement is not able to protect hepatocytes from CCl_4_-induced acute injury. In fact, we found that AAV8-mediated GNMT expression was downregulated due to the loss of AAV8-GNMT transduced hepatocytes seen in severe damage (data not shown). Notably, significantly reduced ALT and AST levels were observed in the AAV8-GNMT group in the latter stage of CCl_4_ treatment, and furthermore, the AAV8-GNMT vector diminished CCl_4_-induced liver fibrosis. These sets of evidence suggests that the protective ability of GNMT overexpression might relate to the downregulation of liver fibrogenesis and the enhancement of hepatocyte proliferation.

Regulation of hepatic fibrosis and hepatocyte proliferation involves crosstalk between non-parenchymal and parenchymal cells. Advancing hepatic fibrosis inhibits the progression of hepatocyte proliferation, and the deterioration of hepatocyte proliferation leads to replacement of the normal parenchyma by fibrotic matrix^46^. TGF-β1 is not only a pivotal profibrogenic cytokine in liver fibrosis, but also an inhibitor of hepatocyte proliferation. Ebrahimkhani *et al*. demonstrated that activated HSCs suppress hepatocyte proliferation by generating TGF-β1, suggesting that activated HSCs in the diseased liver are negative regulators of hepatocyte regeneration^46^. In our study, CCl_4_-induced fibrosis was attenuated in AAV8-GNMT group as seen by the downregulation of TGF-β1, collagen I and α-SMA. High levels of TGF-β1 resulting from chronic liver damage may lead to massive hepatocyte cell apoptosis via stimulation of TGFβ-SMAD signaling pathway, which in turn may exacerbate activation of HSCs and development of liver fibrosis^47^. Therefore, exogenous AAV8-GNMT may interfere with the TGFβ-SMAD signaling pathway in hepatocytes and thus prevent hepatocyte apoptosis and HSC activation and promote hepatocyte regeneration. Pretreatment of AAV8-GNMT upregulated expression of Ki-67 one day after the first CCl_4_ injection, indicating AAV8-GNMT promotes hepatocyte regeneration upon liver injury. It remains unclear how AAV8-GNMT promotes liver regeneration. Recent studies have shown that liver regeneration is impaired in GNMT knockout mice, and SAM plays a crucial role in hepatocyte growth^48^. Liver SAM is 50-fold higher in GNMT depleted mice than in WT and it has been shown that decreased level of SAM is essential for the initiation of normal regeneration after partial hepatectomy^49^. SAM blocks hepatocyte growth factor (HGF)-induced DNA synthesis via inhibition of the LKB1/AMP-activated protein kinase (AMPK)/endothelial nitric oxide synthase (eNOS) cascade^50^. These reports suggest that AAV8-GNMT may be involved in the regulation of SAM, leading to HGF-mediated mitogenic effects in hepatocytes.

In the present study, we demonstrated for the first time the *in vivo* correction of diverse liver disease models by gene therapy using a recombinant AAV8 vector containing human GNMT cDNA. AAV8-GNMT expression provided long-term protection and partially compensated the effects of GNMT deficiency. The phenomenon that AAV8-GNMT inhibited liver inflammation and tumor growth in *Gnmt*^−/−^ mice suggested therapeutic potential of AAV8-GNMT in such patients with congenital GNMT deficiencies. In addition, AAV8-GNMT ameliorated CCl_4_-induced liver fibrosis, suggesting that AAV8-mediated GNMT intervention may have beneficial effects on chronic liver diseases. Therefore, the AAV8-GNMT vector can be a potential method for future development of liver protection. For advance understanding of the protective and therapeutic function of AAV8-mediated GNMT vector, other chronic animal models, such as mouse models for HBx TG and non-alcoholic fatty liver disease, can be used for further investigation.

## Materials and Methods

### Preparation of AAV8-GNMT and AAV8-eGFP

The pAAV8-eGFP plasmid containing the CMV promoter and the eGFP coding sequence (CDS) was provided by Dr. Mi-Hua Tao (Institute of Biomedical Sciences, Academia Sinica, Taiwan). The eGFP CDS was replaced with the human GNMT CDS fragment in pAAV8 backbone by EcoRI and XhoI. These plasmids were amplified and extracted by Qiagen mega plasmid purification kit (Qiagen, Hilden, Germany). The AAV8 vector carrying eGFP or GNMT CDS was prepared by using the AAV Helper- Free System, a gift from Dr. Mi-Hua Tao^13^. Briefly, pAAV8-eGFP and pAAV8-GNMT were co-transfected with pHelper and pXX8 into 293 cells, respectively, by a calcium phosphate-based protocol. Viral particles were harvested following 48 h of transfection. After purification by CsCl gradient centrifugation, the titers of viral particles were determined by quantitative real-time PCR and these vectors were applied in the following animal experiments.

### Animal experiment

C57BL/6 and BALB/c mice were purchased from the National Laboratory Animal Center in Taiwan. C57BL/6 mice were used to generate the *Gnmt*^−/−^ strain, which has been described by Liu *et al*.^25^. All animal experiments were carried out in accordance with the Guide for the Care and Use of Laboratory Animals reviewed and approved by the Institutional Animal Care and Use Committee of Kaohsiung Medical University. The study was performed according to the guidelines and regulations. Four-month-old female *Gnmt*^−/−^ (KO) mice were divided into three groups: KO (n = 6), KO-AAV8-GNMT (n = 6) and KO-AAV8-eGFP controls (n = 5). Mice were given AAV8-GNMT (1 × 10^11^ viral genomes in 100 μl saline) or AAV8-eGFP (1 × 10^11^ viral genomes in 100 μl saline) by intravenous injection (Fig. 1a). In the CCl_4_-induced fibrosis mouse model, BALB/c wild-type mice were divided into four groups: normal control (n = 8), AAV8-GNMT/CCl_4_ (n = 7), AAV8-eGFP/CCl_4_ (n = 8), and CCl_4_ (n = 10). These groups of mice were given AAV8-GNMT (1 × 10^11^ viral genomes in 100 μl saline), AAV8-eGFP (1 × 10^11^ viral genomes in 100 μl saline) or the equal volume of saline by intravenous injection. After 2 weeks of AAV8 injection, mice were treated intraperitoneally with corn oil or CCl_4_ (1 ml/kg body weight dissolved in corn oil) twice a week for 8 weeks. Ultrasound imaging and analysis of blood biochemistry were performed to monitor the progression of liver injury and tumor formation during the study period until sacrifice.

### Blood biochemical analysis

Blood was collected after depriving mice of food for 12 hours by tail artery sampling. Serum samples were frozen at −80 °C until assayed. Serum ALT, AST, triglyceride and cholesterol levels were measured by using FUJI DRI-CHEM NX500i analyzer (Fujifilm, Tokyo, Japan).

### Enzyme-Linked Immunosorbent Assay (ELISA)

The levels of TNF-α and IL-6 in mice serum were detected by commercial ELISA kits (Arigo biolaboratories, Hsinchu, Taiwan). Expression of AFP was measured using a mouse Quantikine ELISA kit (R&D Systems, Minneapolis MN). These assays were manipulated according to the manufacturer’s protocols and instructions.

### Ultrasonography

For ultrasound imaging, the Vevo 2100 high-frequency ultrasound system (Fujifilm VisualSonics, Toronto, Canada) was used in this study. Mice were anesthetized in supine position on a heated stage under 2.5% isoflurane inhalation in oxygen. Abdominal hair was removed prior to sonography to avoid imaging artefact. Sonographic examinations were performed in fundamental brightness mode (B-mode) with the MS-550D probe under the following settings: frequency, 40 MHz; dynamic range, 22 dB; frame rate, 28 frames per second; depth, 15 mm; width, 14.1 mm and transmit power, 100%. Tumor size was determined by the formula: tumor volume [mm^3^] = (length [mm]) × (width [mm])^2^ × 0.52. Based on the study by Lessa *et al*., liver fibrosis/cirrhosis was identified by the ultrasonographic scanning as increased echogenicity and irregular or nodular hepatic surface^51^.

### Real-time PCR

Total RNA was isolated from mouse liver by using the TRIzol^®^ (Invitrogen, Carlsbad, CA) in accordance with the manufacturer’s instructions. Three micrograms of RNA were reverse-transcribed by using Tetro cDNA Synthesis kit (BIOLINE, Taunton, Massachusetts). The real-time PCR reactions were performed by using KAPA SYBR^®^ FAST qPCR Kits (Kapa Biosystems, Wilmington, Massachusetts). Primer sequences are listed in Table 1.

**Table 1:** Primer sequences.

Gene name	Species	Sequence
eGFP	Firefly	F: 5′- CACCATCTTCTTCAAGGACGA-3′
		R: 5′- TGATGCCGTTCTTCTGC-3′
GNMT	Human	F: 5′- ACTGGATGACTCTGGACAA-3′
		R: 5′- ACTGAGGATGTGGTCGT-3′
GNMT	Mouse	F: 5′- GTTGACGCTGGACAAAGA-3′
		R: 5′- AGCCTGTGCTGAGGATA −3′
F4/80	Mouse	F: 5′- CAAGACTGACAACCAGACG-3′
		R: 5′- ACAGAAGCAGAGATTATGACC-3′
TNF-α	Mouse	F: 5′- GCCTCTTCTCATTCCTGCTTG-3′
		R: 5′- CTGATGAGAGGGAGGCCATT-3′
IL-6	Mouse	F: 5′- GGACTGATGCTGGTGAC-3′
		R: 5′- CATTTCTTTGTATCTCTGGAAGTT-3′
Collagen I	Mouse	F: 5′- AAGAGGCGAGAGAGGTT-3′
		R: 5′- CCTTTGGGACCAGCATC-3′
TIMP1	Mouse	F: 5′- CACAAGTCCCAGAACCG-3′
		R: 5′- GTCCACAAACAGTGAGTGTC-3′
α-SMA	Mouse	F: 5′- ATTCAGGCTGTGCTGTC-3′
		R: 5′- TCTCACGCTCGGCAGTA-3′
Desmin	Mouse	F: 5′- CAGGCAGCCAATAAGAAC-3′
		R: 5′- GCCATCTCATCCTTTAGGT-3′
TGF-β1	Mouse	F: 5′- CCAAAGACATCTCACACAGTA-3′
		R: 5′- GCCACTCAGGCGTATCA-3′
MMP13	Mouse	F: 5′- TGCCATTACCAGTCTCCG-3′
		R: 5′- TGTCATAACCATTCAGAGCCC-3′
β-actin	Mouse	F: 5′- TGCTCGGGACGTTCACAAC-3′
		R: 5′- GAGAATAAAGCAACTGCACAAACAA-3′

### Western blotting

Culture cells were lysed by using RIPA lysis buffer supplemented with protease inhibitor cocktail (Roche, Mannheim, Germany). Proteins were subjected to SDS-PAGE and separated by 10% resolving gel. The primary antibodies used in the experiment included anti-flag and anti-β-actin (Sigma-Aldrich, Munich, Germany).

### Histopathological examination

Liver tissues were fixed in 10% formalin, embedded in paraffin, consecutively sectioned with a thickness of 5 μm, mounted and heat-fixed on glass slides. H&E staining was performed according to the standard protocol by Taiwan Mouse Clinic (National Comprehensive Mouse Phenotyping and Drug Testing Center, Taipei, Taiwan). Collagen deposition was evaluated by Masson’s trichrome staining (HT15-1KT, Sigma-Aldrich).

### Immunohistochemical staining

Tissue sections were deparaffinized and subsequent blockage of the endogenous peroxidase activity was achieved by incubation with 2.5% methanolic hydrogen peroxide for 30 min. The tissue sections were stained with rabbit antibodies against Ki-67, MMP13, collagen I, desmin (GeneTex, Irvine,CA) or TIMP-1 (Proteintech Group Inc, Chicago, USA) by using the Mouse/Rabbit Probe HRP Labeling Kit (BioTnA Biotech, Kaohsiung, Taiwan). Binding of mouse monoclonal antibodies against GNMT (14-1, YMAC Bio Tech, Taiwan), α-SMA (Lab Vision, Fremont, CA) and TGF-β1 (R&D Systems) was detected by using the N-Histofine Mousestain Kit (Nichirei Biosciences, Tokyo, Japan). The immunohistochemical assays were performed according to the manufacturer’s instructions. The sections were developed with 3-3′ diaminobenzidine (DAB) (Dako, Milan, Italy) and finally counterstained with hematoxylin (Dako). Negative controls were performed using normal mouse antiserum instead of the primary antibody.

### Statistical analysis

All experiments were performed independently and repeated at least three times. Data from the experiments were shown as mean ± standard error of the mean (SEM). Statistical comparisons were evaluated by the student t test as well as the one-way ANOVA test. Tumor incidence was analyzed by the Mann–Whitney U-test. P < 0.05 was considered to be statistically significant.

## Electronic supplementary material

Supplementary information
