# Supplementary material for: AAV serotype 8-mediated liver specific GNMT expression delays progression of hepatocellular carcinoma and prevents carbon tetrachloride-induced liver damage
Source: Sci Rep. 2018 Sep 14;8:13802. doi: 10.1038/s41598-018-30800-3 (PMC6138656; doi:10.1038/s41598-018-30800-3)

Supplementary information

AAV serotype 8-mediated liver specific GNMT expression delays progression of hepatocellular carcinoma and prevents carbon tetrachloride-induced liver damage

Cheng-Chieh Fang[δ1], Ching-Fen Wu[δ1], Yi-Jen Liao[2], Shiu-Feng Huang[3], Marcelo Chen[4,5], Yi-Ming Arthur Chen*[1,6,7]

[1]Center for Infectious Disease and Cancer Research (CICAR), Kaohsiung Medical University, Kaohsiung, Taiwan;

[2]National Mosquito-Borne Diseases Control Research Center, National Health Research Institutes, Miaoli, Taiwan;

[3]School of Medical Laboratory Science and Biotechnology, College of Medical Science and Technology, Taipei Medical University, Taipei, Taiwan;

[4]Institute of Molecular and Genomic Medicine, National Health Research Institutes, Miaoli, Taiwan;

[5]Department of Urology, Mackay Memorial Hospital Taipei, Taiwan;

[6]School of Medicine, Mackay Medical College, New Taipei City, Taiwan;

[7]Institute of Biomedical Sciences, National Sun Yat-sen University, Kaohsiung, Taiwan;

[8]Department of Microbiology and Immunology, Institute of Medical Research and Institute of Clinical Medicine, College of Medicine, Kaohsiung Medical University, Kaohsiung, Taiwan.

***Correspondence should be addressed to** Yi-Ming Arthur Chen (E-mail: arthur@kmu.edu.tw), No. 100, Shih-Chuan 1st Rd, Kaohsiung City, Taiwan 80708. Phone: 886-7-3117820; Fax: 886-7-3212062

δ: These authors contributed equally to this work.

Figure S1. Original WB image for Fig. 1c

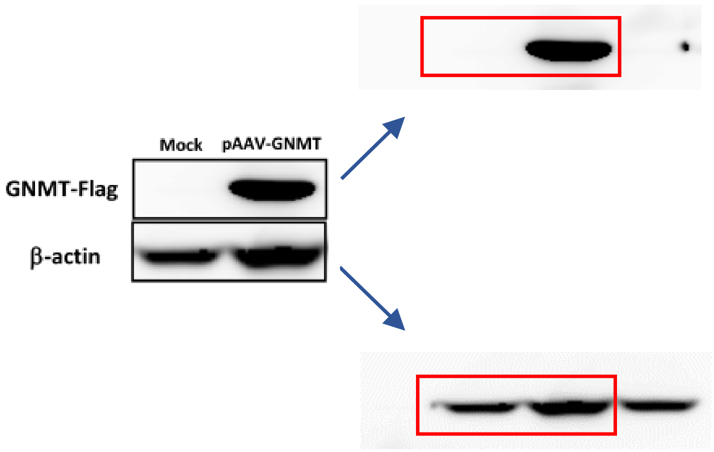

Supplement: Supplementary file 1 — Supplementary information [file 41598_2018_30800_MOESM1_ESM.pdf]
